# Numerical Analysis of Curing Residual Stress and Strain in NEPE Propellant Grain

**DOI:** 10.3390/polym15041019

**Published:** 2023-02-17

**Authors:** Xiangyang Liu, Xuyuan Xie, Dongmo Zhou, Ruimin Wang

**Affiliations:** 1 School of Mechatronic Engineering, North University of China, Taiyuan 030051, China; 2 School of Aerospace Engineering, Beijing Institute of Technology, Beijing 100081, China

**Keywords:** curing, residual stress, residual strain, NEPE propellant, chemical shrinkage

## Abstract

In order to investigate the formation mechanism of the residual stress and residual strain in a nitrate ester plasticized polyether (NEPE) propellant grain during the curing and cooling process, the temperature, curing degree and stress/strain of the NEPE propellant grain during the curing and cooling process were analyzed via ABAQUS finite element software. The results indicate that there is a temperature gradient in the NEPE propellant grain during curing at 50 °C. The maximum temperature difference is about 5 °C and the maximum temperature is located on the center of propellant grain. At the end of curing, the temperature in the interior of the grain tends to be uniform. The curing degree in the NEPE propellant grain during the curing process has the same trend as temperature. The residual stress/strain of the NEPE propellant grain during the curing and cooling down processes are mainly composed of curing shrinkage stress/strain in the curing process and thermal stress/strain in the cooling down process. The curing shrinkage stress and strain in the curing process account for 19% and 31% of the whole process, respectively. The thermal stress and thermal strain in cooling down process account for 75% and 69% of the whole process, respectively. The thermal stress and thermal strain in the curing process can nearly be ignored. The residual stress and residual strain calculated by the traditional method is larger than that obtained in this paper. The maximum deviation of the residual stress and residual strain are about 8% and 17%, respectively.

## 1. Introduction

NEPE propellant represents a significant breakthrough in high-energy solid propellants. It combines the advantages of both composite propellants and double-base propellants. This means that it has both high energy and good mechanical properties [1]. NEPE propellant has the highest specific impulse among any solid propellants that have been used in solid rocket motors (SRMs). In the preparation of the NEPE propellant grain, the propellant slurry needs to be cast and cured separately at an elevated temperature for the required number of days and then cooled to the room temperature before storage [2]. During the curing process, several phenomena, such as thermal expansion, chemical shrinkage, and differences in the thermal expansion coefficient of materials, can lead to the generation and development of residual stress in the propellant grain, and then a reduction in the mechanical properties of the propellant materials, which is even large enough to crack the propellant grain without mechanical loading [3]. Therefore, it is important to evaluate the residual stresses and strains of the propellant grain in the manufacturing process.

In general, residual stresses and strains in a propellant grain of case-bonded SRM are primarily generated by four effects [4]:

- Thermal expansion and contraction during the curing process;
- Different thermal expansion coefficients between propellant and case;
- Difference between curing temperature and operating temperature of SRMs;
- Chemical shrinkage of the propellant during the curing process.

For composite materials such as composite solid propellant, the overall residual stress introduced from curing in previous studies was mainly determined by considering two contributions: the thermal expansion and contraction of propellant cooling from the curing temperature to room temperature and the chemical shrinkage of matrix resin from the crosslink polymerization during curing [5]. It was found that thermal expansion and contraction during the curing and cooling down processes was the most significant factor in the generation of residual stresses [6]. This involves the first three aforementioned factors, which have been extensively investigated using the finite element method (FEM) due to its significant economy, efficiency and accuracy [7,8,9]. However, chemical shrinkage cannot be neglected; the research shows that the residual stresses due to chemical shrinkage may contribute up to 30% of the total residual stresses in composites [10]. The effect of chemical shrinkage on residual stresses in propellant grain is usually converted into a temperature effect. For composite solid propellant, the conversion temperature is usually 8 °C; that is, when the curing temperature is 50 °C, the stress-free temperature of propellant grain is 58 °C [11,12,13]. The essence of this method is to use thermal expansion to compensate for some of the chemical shrinkage that occurs during the curing process. Unfortunately, the chemical shrinkage process of propellant during the curing process is rarely considered, and the formation mechanism of residual stress in the propellant grain is still unclear.

In the curing process, the crosslinking reaction of the adhesive system is induced between a prepolymer and curing agent. The final crosslinking structures present some new bonds and the molecular growth continues over time until a perceptible gel-like lump can be formed; this also resulted in volume shrinkage of the adhesive system [14]. The point at which the adhesive system is converted from the liquid phase to the solid phase is called the gel point [15]. Typically, most propellants begin to shrink before the gel point, but little or no stress is developed in the resin before the gel point, because modulus development is minimal at cure states below the gel point [16,17]. The modulus of the propellant increases rapidly after the gel point and there is still some volume shrinkage, since the residual stress level mainly depends upon the product of the volumetric shrinkage and the stiffness of the propellant material [18], and a considerable residual stress will be introduced. The curing shrinkage stress should be paid enough attention in the residual stress analysis of the propellant grain.

Therefore, the residual stress and strain of an NEPE propellant grain during the entire curing and cooling processes was investigated in this paper. The organization of the paper is as follows. The theoretical framework for analyzing the residual stress and strain of the NEPE propellant is constructed in Section 2. The finite element modeling of the numerical simulation is established in Section 3. The residual stress and strain in the NEPE propellant grain is analyzed in Section 4. Finally, the conclusions are summarized in Section 5.

## 2. Theoretical Models

### 2.1. Thermo-Chemical Model

The thermo-chemical model is composed of heat conduction and cure kinetics. The temperature field of the propellant grain depends on the external curing temperature profile and the heat released by the curing reaction of the propellant, which is considered as a non-linear temperature transfer problem with a heat source. The three-dimensional heat transfer equation of the isotropic composites during a curing process can be expressed as [19]:

(1) $$\rho C_{p}\frac{\partial T}{\partial t}=\frac{\partial }{\partial x}\left(k\frac{\partial T}{\partial x}\right)+\frac{\partial }{\partial y}\left(k\frac{\partial T}{\partial y}\right)+\frac{\partial }{\partial z}\left(k\frac{\partial T}{\partial z}\right)+\frac{\partial Q}{\partial t}$$

where ρ is the propellant density; $C_{p}$ is the specific heat capacity of propellant; t is the absolute time; T is the transient temperature of the propellant at time t; k is the thermal conductivity; and Q is the internal heat source, which can be expressed by the following equation:

(2) $$\frac{\partial Q}{\partial t}=\rho H_{r}\frac{d\alpha }{dt}$$

where α and dα/dt are the cure degree and the curing rate of the propellant, respectively, and $H_{r}$ is the total release heat during curing.

For the cure reaction of an NEPE adhesive system, its cure kinetic is typically described using a cure kinetic equation with an Arrhenius-type temperature dependency. An example is the well-known Kamal–Sourour autocatalytic cure kinetic model expressed as in [20]:

(3) $$\frac{d\alpha }{dt}=A_{0}\exp(-E_{a}/RT)\alpha^{m}{\left(1-\alpha \right)}^{n}$$

where $A_{0}$ is the pre-exponential factor, $E_{a}$ is the activation energy, and m and n are reaction orders.

The boundary condition used in this study is the third-type boundary condition (convection), which indicates the convection heat transfer between the boundaries of the solution range and the ambient temperature, which can be expressed as the following [21]:

(4) $$k\left(\frac{\partial T}{\partial x}n_{x}+\frac{\partial T}{\partial y}n_{y}+\frac{\partial T}{\partial z}n_{z}\right)=h(T_{a}-T)$$

where h is the convective heat transfer coefficient. The natural convection heat transfer coefficient is usually estimated to be 10 $W/m^{2}\cdot K$ and $T_{a}$ is the ambient temperature.

### 2.2. The Macro Residual Stresses and Strains Model

The residual strain comprises the sum of the thermal strain and chemical shrinkage strain throughout the curing and cooling process. Thus, the strain relation can be defined as [22]:

(5) $$\varepsilon^{tot}=\varepsilon^{th}+\varepsilon^{sh}$$

where $\varepsilon^{tot}$, $\varepsilon^{th}$, and $\varepsilon^{sh}$ are the total residual strain, thermal strain, and chemical shrinkage strain, respectively.

As the propellant cures, it also undergoes a volumetric shrinkage that is associated with the reaction process. This volumetric shrinkage manifests itself into a chemically induced contraction strain in the propellant. For a given incremental change in the curing degree Δα during the reaction, the associated incremental change in specific volume $\Delta V_{sh}$ of the resin can be expressed according to [23]:

(6) $$\Delta V_{sh}=\Delta \alpha \cdot V_{sh}$$

where $V_{sh}$ is the total volume change in the adhesive system at full cure. The strain contraction in all directions is assumed to be equal. The incremental isotropic shrinkage strain of $\Delta \varepsilon^{sh}$ and $\Delta V_{sh}$ can be related by [23]:

(7) $$\Delta \varepsilon^{sh}=\sqrt[{3}]{1+\Delta V_{sh}}-1$$

The cure shrinkage strain in the resin during the curing process is the cumulative sum of all the incremental contributions, as determined through Equations (6) and (7). The solid particles themselves are assumed not to undergo any chemical contraction during the curing process.

The thermal expansion behavior of the propellant is assumed to be independent of the degree of curing and follows the usual linear relationship with the temperature. The incremental change in the thermal strain $\Delta \varepsilon^{th}$ in the propellant grain caused by an incremental change in the temperature ΔT can be expressed as:

(8) $$\Delta \varepsilon^{th}=\gamma \cdot \Delta T$$

where γ is the thermal expansion coefficient of the propellant.

During the NEPE propellant production, the propellant is fully cured at 50 °C for 7 days, then is gradually cooled to room temperature [24]. Thus, the constitutive behavior of the NEPE propellant can be decomposed into two parts. The first step is the curing stage; the propellant modulus is a linear elastic model that increases with the change in the curing degree. The second step is the cooling stage where propellants are regarded as viscoelastic materials. With the help of user subroutine UMAT in ABAQUS software platform (ABAQUS ver. 2020, Simulia; Dassault Systemes, France), the two-stage constitutive combination is realized and the numerical analysis is completed [25].

The incremental stress and strain in the first step are calculated as:

(9) $$\sigma =\sum_{i=1}^{N}{\left\{\Delta \sigma \right\}}_{i}=\sum_{i=1}^{N}{\left\{C\right\}}_{i}{\left\{\Delta \varepsilon \right\}}_{i}$$

where N is a specified incremental time step, ${\left\{\Delta \sigma \right\}}_{i}$ and ${\left\{\Delta \varepsilon \right\}}_{i}$ are the stress and strain at each time step i, and C is the stiffness matrix of the propellant.

In the second step, the general form of the integral constitutive equation for the three-dimensional linear viscoelastic materials is as follows:

(10) $$\sigma_{i}(t)=\int_{0}^{t}C_{ij}(t-t^{\prime })\frac{\partial \varepsilon_{j}}{\partial t^{\prime }}d\tau$$

where $\sigma_{i}$ and $\varepsilon_{i}$ denote the stress tensor and strain vectors, respectively. $C_{ij}$ is the relaxed stiffness matrix. t and $t^{\prime }$, respectively, represent the current time and dummy time integration variable. Equation (10) is applicable to isothermal conditions. For a linear orthotropic viscoelastic constitutive law under the curing process or changeable temperature where the material stiffness varies with the temperature and degree of cure, it can be expressed in the following form with the time-temperature equivalence principle:

(11) $$\sigma_{i}(t)=\int_{-\infty }^{t}G_{ij}(\xi -\xi^{\prime })\frac{\partial \varepsilon_{j}}{\partial \xi^{\prime }}d\xi^{\prime }$$

where ξ and $\xi^{\prime }$ are the current and past reduced time, respectively. They are the function of the degree of cure α and temperature *T*, and are given by:

(12) $$\left\{ \begin{array}{l} \xi =\xi (t)=\int_{0}^{t}\frac{dt^{\prime }}{\alpha_{T}[T(t^{\prime })]}\\ \xi^{\prime }=\xi^{\prime }(t)=\int_{0}^{t}\frac{dt^{\prime }}{\alpha_{T}[T(t^{\prime })]} \end{array}\right.$$

where, $\alpha_{T}$ is the displacement conversion factor, which can be described by the following WLF equation:

(13) $$\mathrm{lg}\alpha_{T}=\frac{-C_{1}(T-T_{r})}{C_{2}+(T-T_{r})}$$

where $C_{1}$ and $C_{2}$ are material constants, which can be determined by experiment, T is the current moment temperature, and $T_{r}$ is the reference temperature.

## 3. Finite Element Modeling

### 3.1. Simulation Flow

**Figure 1:**
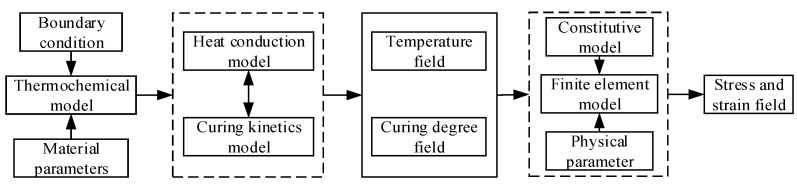
Calculation diagram of numerical simulation.

The curing and cooling behavior of the propellant was simulated using a sequentially coupled formulation based on the ABAQUS software platform. Firstly, the thermal chemical model was used to simulate the heat generation and heat transfer process of the propellant grain during curing and cooling, and the temperature and curing degree of each node were obtained. Then, the thermal mechanical model was introduced to investigate the residual stress and strain of the propellant grain. Within the time range of the curing process, the constitutive with a variable linear elastic model was adopted, and the viscoelastic constitutive was activated for calculation during the cooling down process. The procedure of the simulation model for residual stress and strain is presented in Figure 1.

Based on the kinetic equation of the curing reaction, the constitutive mechanical model and the heat transfer equation, the 3D SRM grain model can be introduced into the user-defined heat release (HETVAL) to simulate the chemical evolution process of the curing, and the curing degree can be obtained. Combined with the user-defined material expansion (UEXPAN), user-defined material mechanical behavior (UMAT), and user-defined field (USDFLD) subroutines, the model can be put into material modulus changes and cure shrinkage, which is capable of calculating the curing degree, deformation, and residual stress of propellant grain [26].

### 3.2. Finite Element Modeling

**Figure 2:**
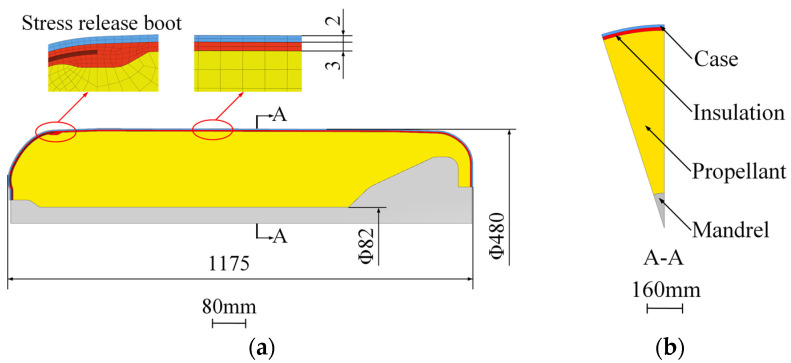
SRM model and mesh. (**a**) 3D SRM model. (**b**) Cross section.

A 3D SRM model (including the core mold) was chosen for this structure in order to predict the stress and strain response in detail. Due to the symmetry of the geometry and loading, a model of an 18° segment with axis-symmetric boundary conditions on the cut faces was utilized for simplicity without loss of accuracy. The NEPE propellant, insulation and case were modeled with 17,808 eight-node solid elements (C3D8R) and 22,933 nodes, as shown in Figure 2.

The model was created with the assumptions and boundary conditions as follows: (i) The thickness of the case was constant. (ii) The insulation liner was elastic. (iii) The outer surface of the case was fixed and subjected to natural convection boundary conditions and the symmetry plane was set with symmetry constraints. (iv) The interfaces between the case/insulation/propellant were set as “Tie” in the ABAQUS software. (v) The interface between the propellant and core mold was given from the assumptions of friction-free contact and subjected to a boundary condition of the first kind.

According to the curing process of the NEPE propellant grain, the calculation conditions were as follows: For Step 1, the curing temperature $T_{C}$ = 50 °C was defined as the initial temperature field and followed by temperature preservation at 50 °C for 7 days. For Step 2, the model cooled slowly from the cure temperature to room temperature (20 °C).

### 3.3. Model Parameters

(i) Cooling down process

The stress relaxation tests of the propellant with the same formulation presented in Ref. [27] were performed based on the traditional uniaxial tension method. The viscoelastic test data were obtained and the viscoelastic behavior in this process can be expressed in the Prony series form as:

(14) $$\begin{array}{ll} E(t) & =0.868+0.561e^{t/0.0002}+0.474e^{t/0.002}\\ & +0.406e^{t/0.02}+0.348e^{t/0.2}+0.298e^{t/2}\\ & +0.255e^{t/20}+0.22e^{t/200}+0.185e^{t/2000} \end{array}$$

where E(t) is the relaxation modulus, MPa.

Meanwhile, the parameters in Equation (13) were obtained as: $C_{1}$ = −7.053, $C_{2}$ = 171.513, $T_{r}$ = 20 °C.

(ii) Curing process

The curing kinetic parameters of the NEPE propellant adopted in this paper are provided in Ref. [27], which are presented in Table 1.

The relaxation modulus and the dynamic storage modulus can be fitted with the Prony series, and the core of the Prony series is the exponential function. The storage modulus at the end of curing in Ref. [27] (831.1 × 10^3^~868.3 × 10^3^ Pa) is approximately equal to the equilibrium modulus (0.868 MPa). In view of the long-term stress relaxation time in the curing process, an empirical dynamic–static modulus conversion equation [15] was adopted to estimate the Young’s modulus of the propellant during the curing process.

(15) $$E_{0}(t)\approx {G^{\prime }(\omega )|}_{\omega =2/(\pi t)}$$

where $E_{0}(t)$ is the Young’s modulus, $G^{\prime }(\omega )$ is the storage modulus, ω is the frequency.

**Figure 3:**
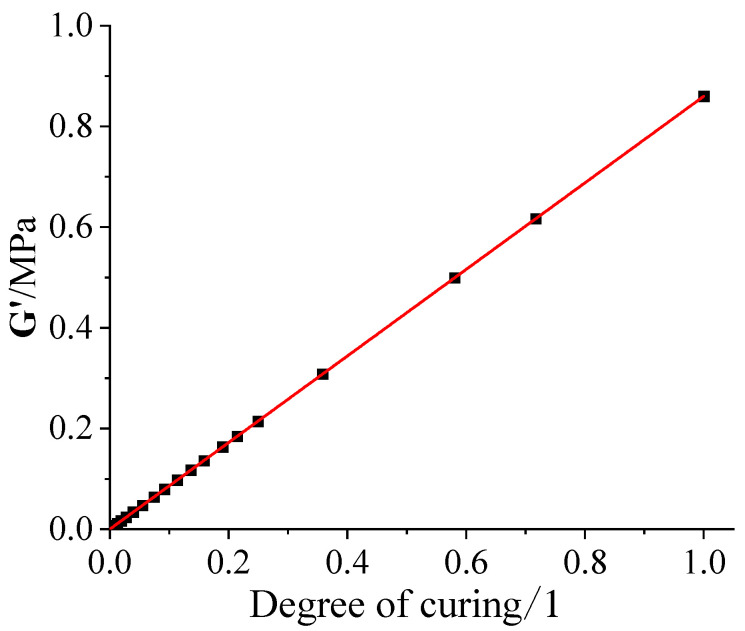
Storage modulus vs. curing degree.

The storage modulus of the NEPE propellant cured at 50 °C with a curing degree provided in the literature [27] is shown in Figure 3.

By fitting the data in Figure 3, the linear elastic modulus of the NEPE propellant during the curing process can be obtained as follows:

(16) $$E_{c}\;=\;61.7\;\times \;10^{5}\alpha$$

where $E_{c}$ is the elastic modulus and α is the curing degree.

The other parameters provided by the manufacturer for numerical analysis are given in Table 2, in which the parameters of the propellant were chosen at the end of curing and their changes during the curing process were ignored.

### 3.4. Modal Verification

**Figure 4:**
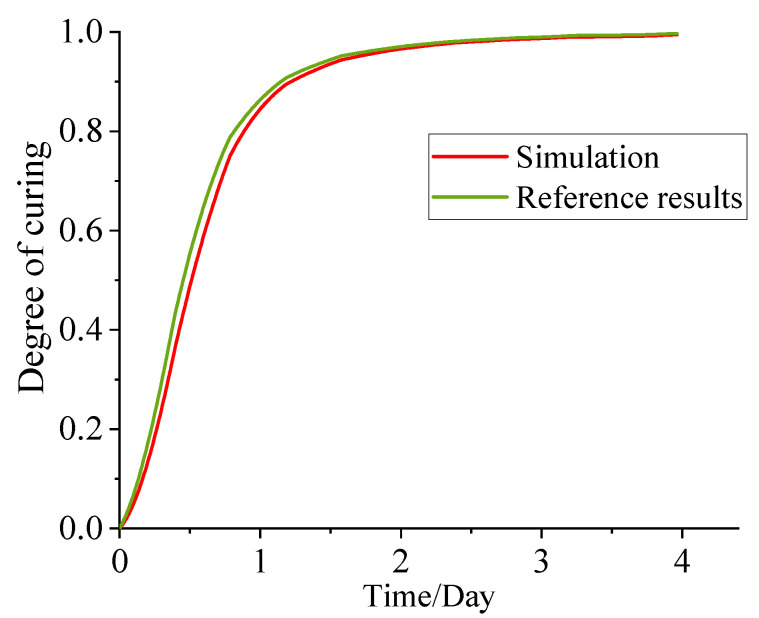
Comparison of the cure degree between the simulation and the result of Ref. [21] at 50 °C.

To check the accuracy of the simulation method in this paper, the FEM simulation result of curing process was compared with the result performed by Rad, H.M. et al. [21], which is based on the numerical method of finite volume. The numerical solution specifications were exactly the same as the conditions in Ref. [21]. Figure 4 compares the cure degree in the center of three-dimensional modeling adopted in the Ref. [21]. It is clear that the results of the cure degree obtained by the two methods are in good agreement and support that the numerical method in this paper reasonably well captures the curing characteristics of the propellant.

## 4. Results and Discussion

### 4.1. Temperature and Curing Degree during Curing Process

**Figure 5:**
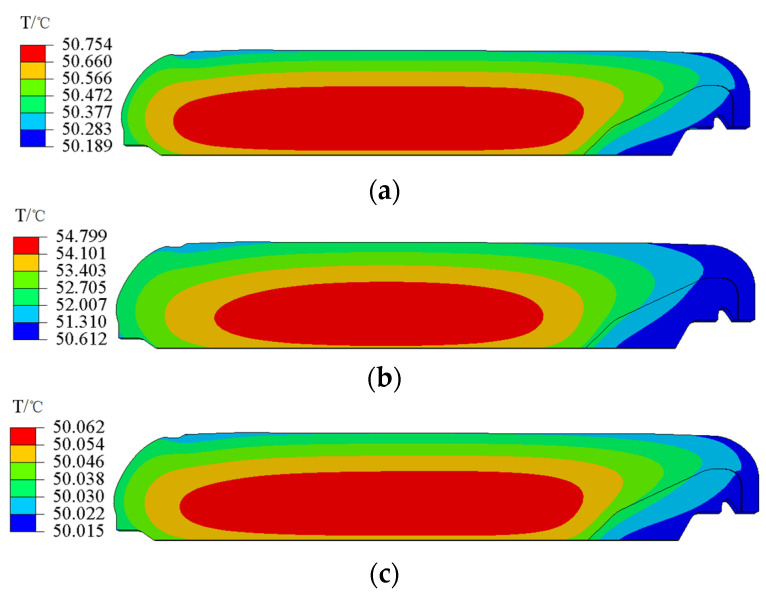
Contours of the temperature. (**a**) 24 h. (**b**) 72 h. (**c**) 168 h.

Figure 5 presents the temperature of the NEPE propellant grain cured at 50 °C for 24 h, 72 h, and 168 h. It can be seen that the temperature in the center of the propellant grain is higher than that in the periphery during the curing process. The temperature in the grain also tends to be uniform at the end of curing (168 h).

**Figure 6:**
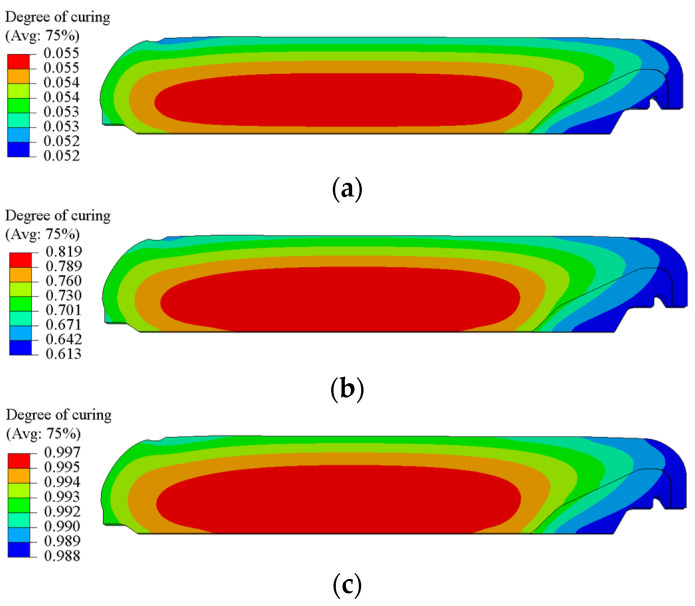
Contours of the curing degree. (**a**) 24 h. (**b**) 72 h. (**c**) 168 h.

Figure 6 depicts the curing degree of the NEPE propellant grain cured at 50 °C for 24 h, 72 h, and 168 h. It can be seen that the curing degree show the same distribution trends as temperature. The curing degree in the center of the propellant grain is higher than that in the periphery. At the end of curing, the curing degree in the interior of the grain tends to be uniform, reaching above 0.997.

**Figure 7:**
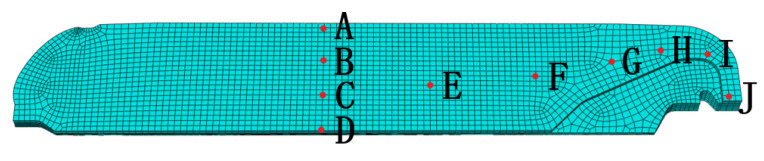
Node selection.

For further analysis of the variation in the characteristics of temperature and curing degree in the NEPE propellant grain during the curing process, 10 nodes along the axial and longitudinal direction, whose gradient changes in temperature and curing degree are obvious, were selected for analysis, as shown in Figure 7.

**Figure 8:**
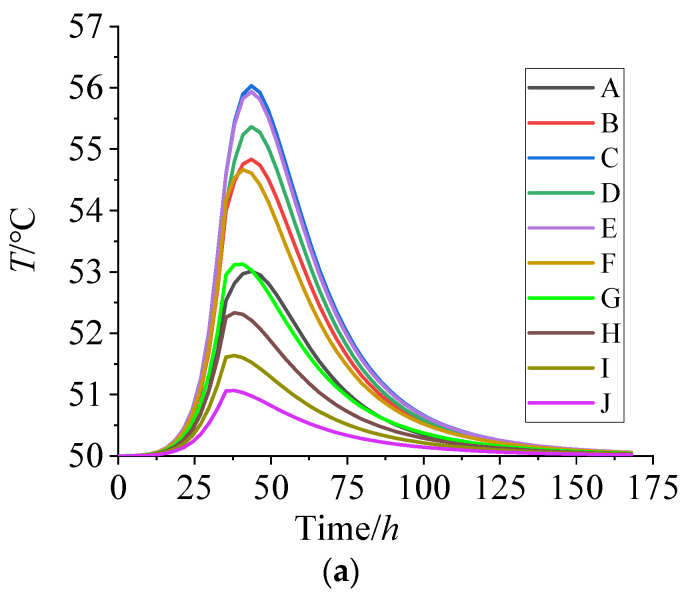

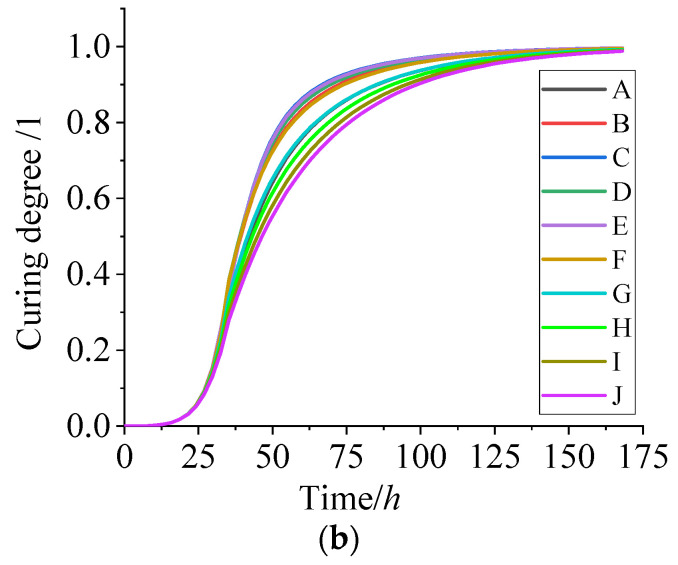
(**a**) Temperature vs. curing time. (**b**) Curing degree vs. curing time.

Figure 8 plots the temperature and curing degree of each node with curing time.

It can be seen from Figure 8a that the temperature of the NEPE propellant grain first increases and later decreases on the whole. The arrival time of the highest temperature increased from 38 h to about 43 h with the node position moved from the periphery to the center of propellant grain. The maximum temperature of grain interior (the nodes of C, E) is 56 °C at the curing time of 43 h. The periphery of the propellant grain, such as the nodes of J, is basically maintained at the curing temperature of 50 °C. After curing for 150 h, the internal temperature of the propellant tends to be uniform, at about 50 °C.

According to Figure 8b, the curing degree of the NEPE propellant increases slowly within 10 h, and then increases quickly and reaches a maximum curing rate at about 32 h. About 50 h later, the curing rate decreases gradually and tends to be stable. The curing degree of the grain interior (the nodes of B, C, D, E, F) is significantly higher than that in the periphery between 35 h and 125 h, but with no significant difference in the other periods.

### 4.2. Residual Stress and Strain of Grain during Curing Process

**Figure 9:**
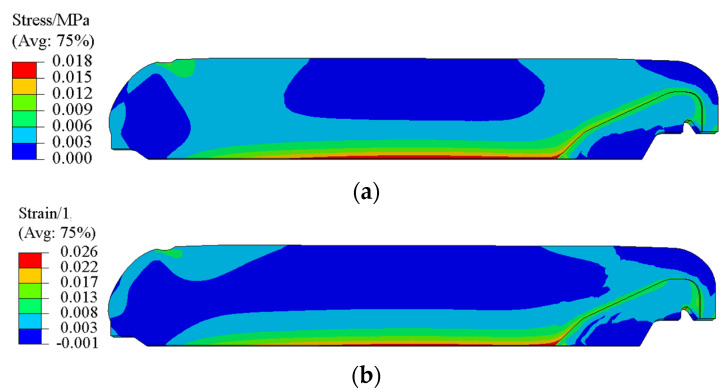
(**a**) Contours of $\sigma_{c}^{tot}$. (**b**) Contours of $\varepsilon_{c}^{tot}$.

The equivalent stress and equivalent strain were adopted to characterize the residual stress and strain of grain. Figure 9 presents the residual stress $\sigma_{c}^{tot}$ and residual strain $\varepsilon_{c}^{tot}$ of the propellant grain during the curing process. It is obvious that the maximum residual stress $\sigma_{c}^{tot}$ and residual strain $\varepsilon_{c}^{tot}$ are located at the inner bore-free surface of the propellant grain, and are 0.018 MPa and 0.026, respectively.

**Figure 10:**
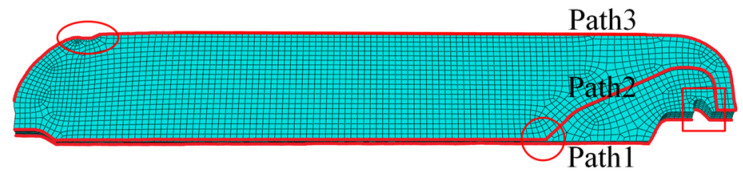
Schematic map of path selection.

To further study the distribution of $\sigma_{c}^{tot}$ and $\varepsilon_{c}^{tot}$ in the propellant grain at the end of curing, three paths as shown in Figure 10 were selected for analysis.

**Figure 11:**
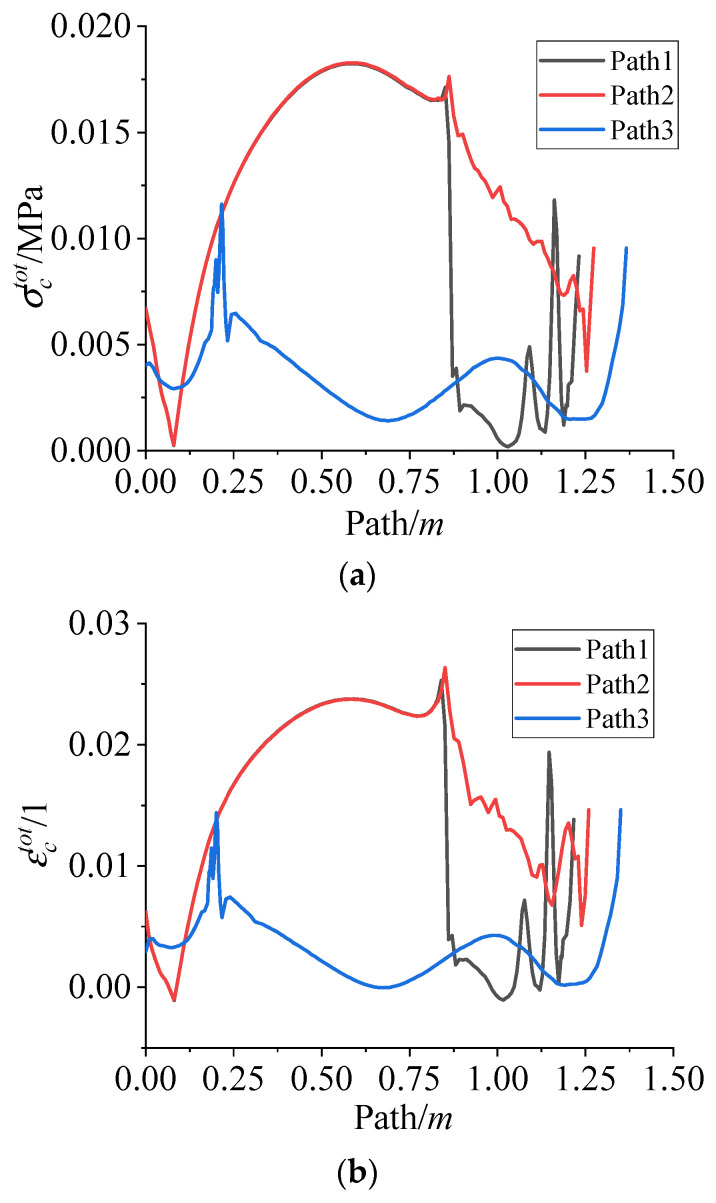
(**a**) The $\sigma_{c}^{tot}$ varies along path 1~3. (**b**) The $\varepsilon_{c}^{tot}$ varies along path 1~3.

Figure 11 presents the $\sigma_{c}^{tot}$ and $\varepsilon_{c}^{tot}$ of the propellant grain along path 1~3. It can be seen that except for the inner bore-free surface of the propellant grain, the stress concentrations still existed in several distinct areas, such as the root of stress release boot (the area marked by the oval in Figure 10) and the junction points between the head of the wing groove and inner bore (the area marked by the circle and square in Figure 10), as marked in Figure 10.

**Figure 12:**
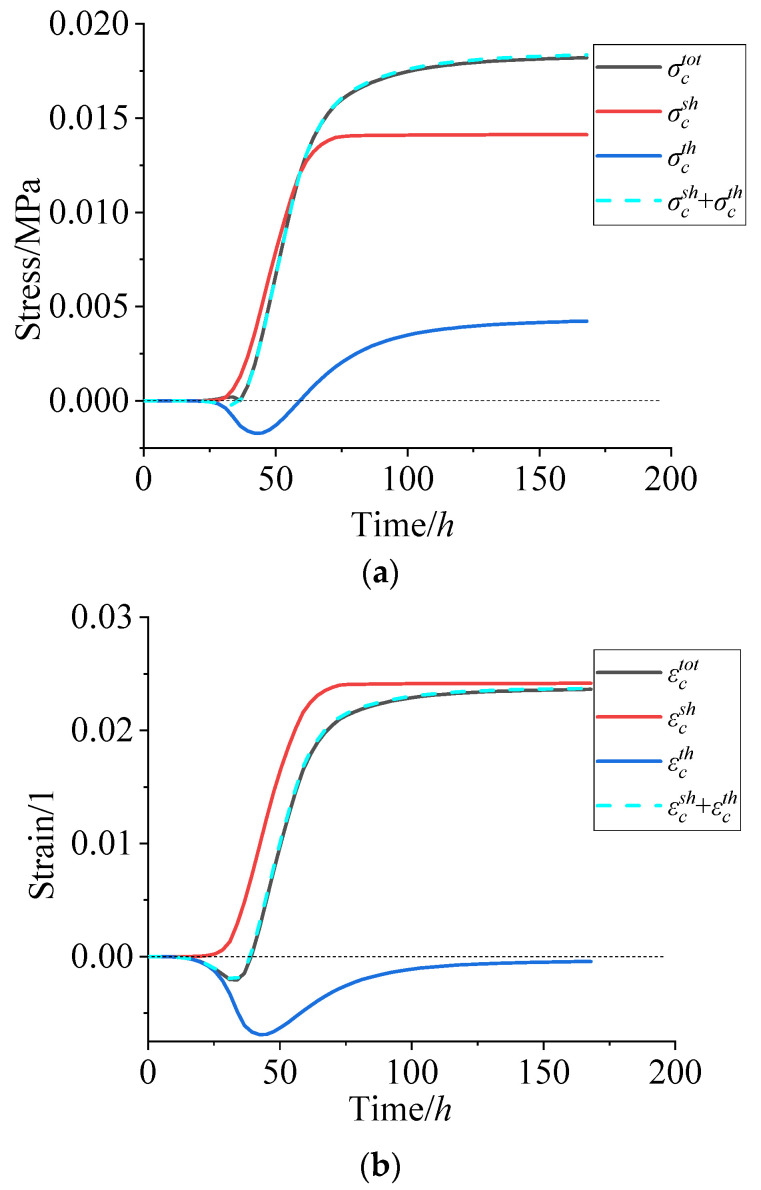
(**a**) Stress vs. curing time. (**b**) Strain vs. curing time.

Since the chemical shrinkage of adhesive system and the thermal cooling contraction of the propellant contributes to the cure residual stress, the $\sigma_{c}^{tot}$ can be expressed as the sum of chemical shrinkage stresses $\sigma_{c}^{sh}$ and thermal stresses $\sigma_{c}^{th}$. In order to further identify the change in three different stresses/strains during the curing process, the three stresses/strains in the maximum residual stress point of path 1 are shown in Figure 12.

According to Figure 12, in the initial 10 h, the 3D cross-linking network of the adhesive system did not obviously form and the propellant slurry was in a viscous flow state, so there was no obvious residual stress generated in the grain. Then, the quick increase in the curing degree was attributed to the chain extension and cross-linking of the molecular chain, and the volume shrinkage and modulus of the propellant also increased quickly, which ultimately resulted in the rapidly increasing $\sigma_{c}^{sh}$ and $\varepsilon_{c}^{sh}$. About 80 h later, the $\sigma_{c}^{sh}$ and $\varepsilon_{c}^{sh}$ increased slowly and finally tended to a certain value. Due to the little change in temperature in the propellant during the curing process, the $\sigma_{c}^{th}$ and $\varepsilon_{c}^{th}$ are smaller on the whole. Since the biggest temperature difference in the propellant grain was about 5 °C at 43 h, the $\varepsilon_{c}^{th}$ reached the maximum value at the same time, accordingly.

Generally, mechanical stretching is considered positive and compression negative [28]. The material in the heated area would result in a conversion of the thermal expansion into compressive strains; thus, the $\varepsilon_{c}^{th}$ is negative during curing process. About 43 h later, the $\sigma_{c}^{th}$ changed from negative to positive. The curing rate of the propellant decreases and the temperature also decreased, the reverse thermal shrinkage of the propellant grain generated reverse thermal stress, together with the modulus being higher during this stage. All of these ultimately resulted in the $\sigma_{c}^{th}$ change from negative to positive.

In addition, according to Figure 12, the sum of $\sigma_{c}^{sh}$ (0.0141 MPa) and $\sigma_{c}^{th}$ (0.0042 MPa) was basically equal to the $\sigma_{c}^{tot}$ (0.0182 MPa) when the curing reaction was completed, and the same as $\varepsilon_{c}^{tot}$, which further verifies that the residual stress and strain in the NEPE propellant grain are superposed by curing shrinkage stress/strain and thermal stress/strain.

### 4.3. Residual Stress and Strain of Grain during Cooling down Process

**Figure 13:**
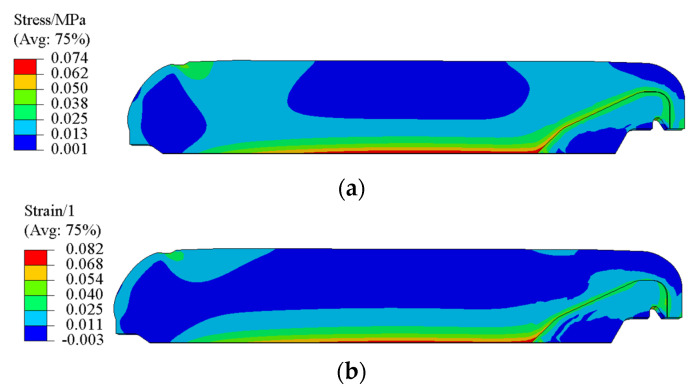
(**a**) Contours of $\sigma^{tot}$ during the cooling process. (**b**) Contours of $\varepsilon^{tot}$ during the cooling process.

After curing, the propellant was taken out and cooled down to room temperature in a dryer. Figure 13 presents the total residual stress $\sigma^{tot}$ and total residual strain $\varepsilon^{tot}$ of propellant grain after cooling down. It is obvious that the $\sigma^{tot}$ and $\varepsilon^{tot}$ were located at the inner bore-free surface of the propellant grain, and were 0.074 MPa and 0.082, respectively.

**Figure 14:**
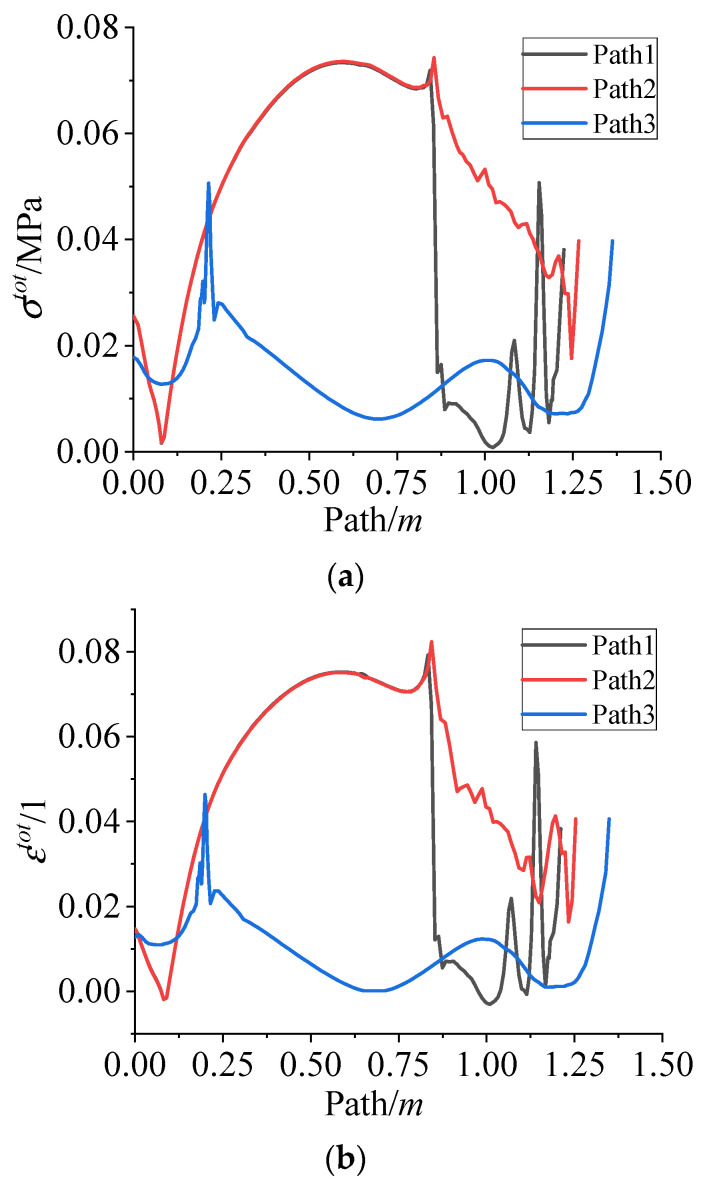
(**a**) The $\sigma^{tot}$ varies along path 1~3 during the cooling down process. (**b**) The $\varepsilon^{tot}$ varies along path 1~3 during the cooling down process.

Figure 14 presents the $\sigma^{tot}$ and $\varepsilon^{tot}$ of the grain along paths 1~3 after cooling. It is clear that the $\sigma^{tot}$ and $\varepsilon^{tot}$ have the same distribution as $\sigma_{c}^{tot}$ and $\varepsilon_{c}^{tot}$, respectively.

**Figure 15:**
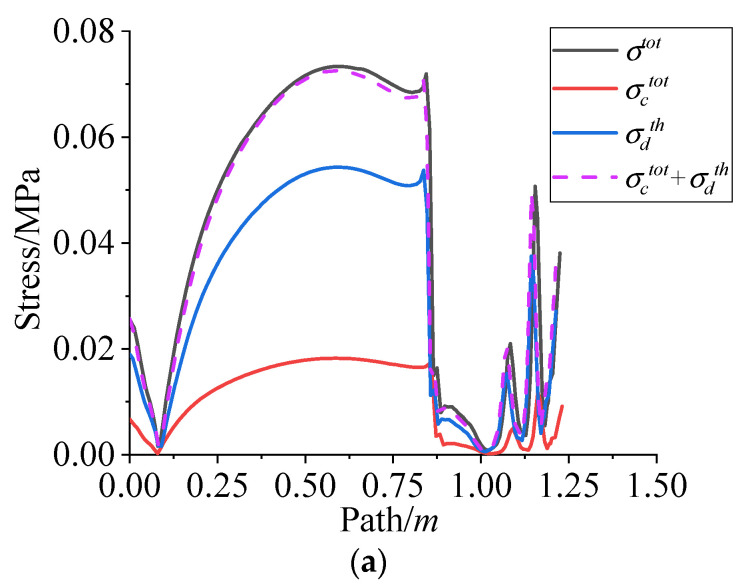

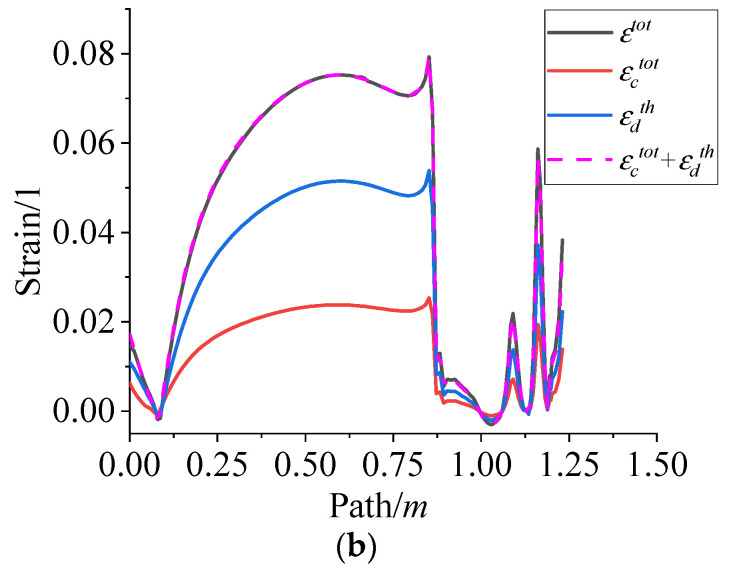
(**a**) Residual stress components vary along path 1. (**b**) Residual strain components vary along the path 1.

After curing and cooling, the components of residual stress/strain on path 1 are shown in Figure 15. It is clear that the sum of $\sigma_{c}^{tot}$ and $\sigma_{d}^{th}$ is basically equal to the $\sigma^{tot}$, and the same as $\varepsilon^{tot}$. That is, during the whole curing and cooling process, the residual stress/strain in NEPE propellant grain is superimposed by the residual stress/strain in the curing stage and the thermal stress/strain in the cooling stage, but the proportion of thermal stress/strain in the cooling stage is higher.

Table 3 shows the proportion of different stresses/strains at the center of path 1 during the curing and cooling down process.

According to Table 3, it can be seen that the total residual stress $\sigma^{tot}$ and total residual strain $\varepsilon^{tot}$ at the central inner hole of the NEPE propellant grain during the curing and cooling down process are mainly caused by the cooling load. The thermal stress and thermal strain during the cooling stage account for 75% and 69% of the whole process, respectively. At the same time, the $\sigma_{c}^{sh}$ and $\varepsilon_{c}^{sh}$ caused by the curing volume shrinkage of the propellant in the curing process cannot be ignored, and both of them account for 19% and 31% of the whole process, respectively. The $\varepsilon_{c}^{th}$ in the curing process can nearly be ignored.

**Figure 16:**
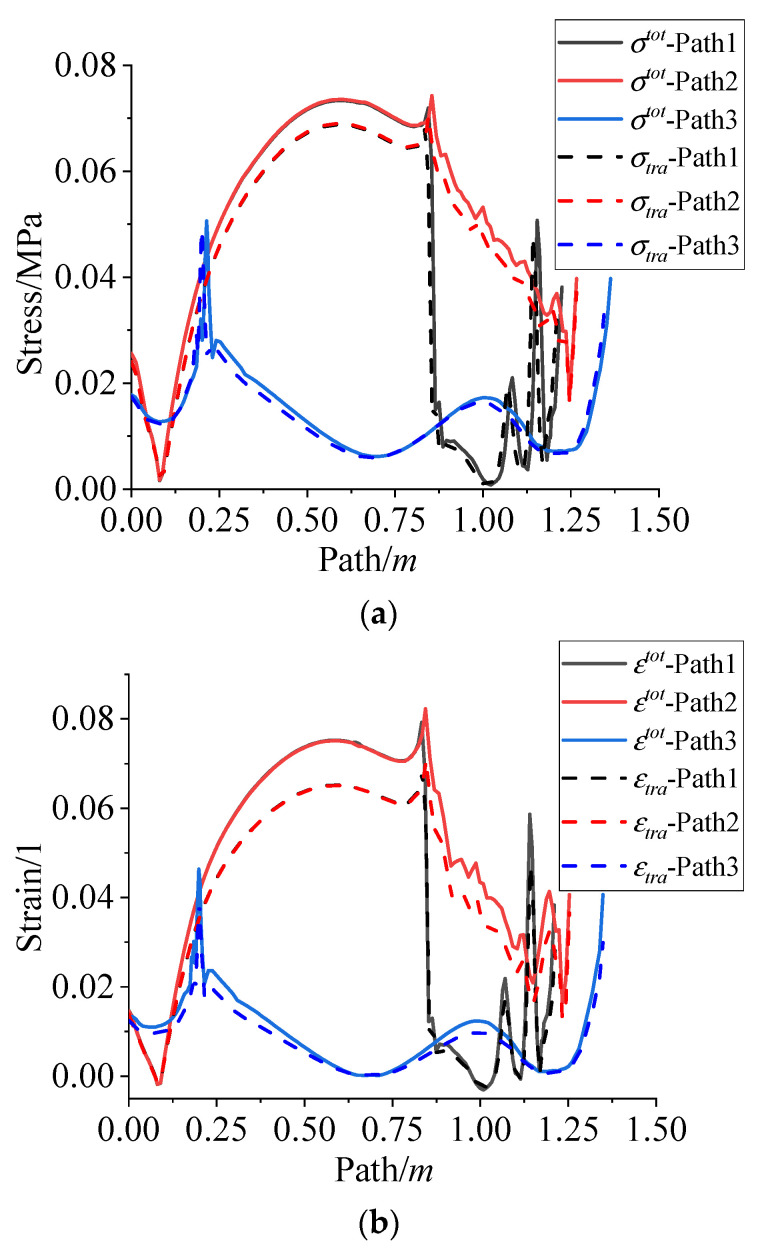
Comparison between the results in this paper and the traditional methods. (**a**) Comparison of residual stress varies along path 1~3. (**b**) Comparison of residual strain varies along path 1~3.

The stress and strain caused by the curing shrinkage of the propellant are usually converted to the equivalent temperature of 8 °C in the traditional way. Figure 16 presents the comparison between the residual stress/strain in this paper and that calculated by traditional methods.

According to Figure 16, it is clear that the residual stress and residual strain calculated by the traditional method are relatively small compared with that obtained in this paper. The maximum deviation of the residual stress and residual strain are about 8% and 17%, respectively. The reason may be that the traditional temperature equivalent conversion method only considers the curing volume shrinkage of the propellant, but does not consider the structural effect of propellant grain. Moreover, there are certain differences in the curing volume shrinkage of different propellants.

## 5. Conclusions

The residual stress and residual strain of the NEPE propellant grain during the curing and cooling down process was investigated through numerical simulation. The conclusions are as follows:

(1) There is a temperature gradient in the NEPE propellant grain during the curing at 50 °C. The maximum temperature difference is about 5 °C and the maximum temperature is located on the center of propellant grain. At the end of curing, the temperature in the interior of the grain tends to be uniform. The curing degree in the NEPE propellant grain during the curing process has the same trend as temperature.
(2) The residual stress/strain of the NEPE propellant grain during the curing and cooling down process are mainly composed of curing shrinkage stress/strain in the curing process and thermal stress/strain in the cooling process. The curing shrinkage stress and strain in the curing process account for 19% and 31% of the whole process, respectively. The thermal stress and thermal strain in the cooling down process account for 75% and 69% of the whole process, respectively. The thermal stress and thermal strain in the curing process can nearly be ignored.
(3) The residual stress and residual strain calculated by the traditional method are smaller than those obtained in this paper. The maximum deviation of the residual stress and residual strain are about 8% and 17%, respectively.

## Figures and Tables

**Table 1:** Curing kinetic parameters of NEPE propellant.

*A*_0_/s^−1^	*E*/(kJ∙mol^−1^)	*m*	*n*	∆H* (kJ∙mol^−1^)
1.241×10^15^	120.6	0.83	1.53	212.34

∆H*: The value was obtained from Eyring model based on the data in Ref. [27].

**Table 2:** Material properties parameters.

Material Parameters	Grain	Insulation	Case
Density/(kg∙m^−3^)	1803	1226	7800
Poisson’s ratio	0.496	0.496	0.3
Expansion coefficient/K^−1^	0.86 × 10^−4^	1.78 × 10^−4^	1.1 × 10^−5^
Heat conductivity/(W∙(m∙K)^−1^)	0.55	0.274	38.95
Specific heat/(J∙(kg∙K)^−1^)	1180	2116	512.91
Elasticity modulus/MPa	-	6.973	210 × 10^3^

**Table 3:** The proportion of residual stress and strain components.

	Value	Component		Value	Proportion
Residual stress	0.074 MPa	Curing stage	$\sigma_{c}^{sh}$	0.014 MPa	19%
			$\sigma_{c}^{th}$	0.004 MPa	6%
		Cooling stage	$\sigma_{d}^{th}$	0.054 MPa	75%
Residual strain	0.082	Curing stage	$\varepsilon_{c}^{sh}$	0.025	31%
			$\varepsilon_{c}^{th}$	−0.00043	0
		Cooling stage	$\varepsilon_{d}^{th}$	0.056	69%

## Data Availability

The data that support the findings of this study are available on request from the corresponding author.
